# Effect of School-Based Educational Intervention on Promoting Healthy Dietary Habits in Danish Schoolchildren: The FOODcamp Case Study

**DOI:** 10.3390/nu15122735

**Published:** 2023-06-13

**Authors:** Malene Outzen, Anne-Vibeke Thorsen, Aleksandra Davydova, Camilla Thyregod, Tue Christensen, Ida Grønborg, Ellen Trolle, Marianne Sabinsky, Gitte Ravn-Haren

**Affiliations:** 1 National Food Institute, Technical University of Denmark, 2800 Kgs. Lyngby, Denmark; 2 DTU Compute, Technical University of Denmark, 2800 Kgs. Lyngby, Denmark; 3 Danish Veterinary and Food Administration, Ministry of Food, Agriculture and Fisheries of Denmark, 2600 Glostrup, Denmark

**Keywords:** intervention study, food camp, healthy dietary habits, food literacy, primary school, nutrition, health promotion

## Abstract

The present study aimed to evaluate the effect of the school-based educational intervention “FOODcamp” on dietary habits among 6th–7th graders (aged 11–13 years), focusing on the food groups: fruits and vegetables, fish, meat, discretionary food, and sugar-sweetened beverages. In this cluster-based quasi-experimental controlled intervention study, 16 intervention classes (322 children) and 16 control classes (267 children) from nine schools were recruited during the school year 2019–2020. The children were asked to record their food intake for four consecutive days (Wednesday to Saturday) before (baseline) and after (follow-up) attending FOODcamp, using a validated self-administered web-based dietary record. Eligible dietary intake registrations from 124 and 118 children from the control and interventions classes, respectively, were included in the final statistical analysis. Hierarchical mixed model analysis was used to evaluate the effect of the intervention. No statistically significant effects of participating in FOODcamp were found on the average food intake of the food groups eaten regularly (vegetables, fruit, vegetables/fruit/juice combined, or meat) (*p* > 0.05). Among the food groups not eaten regularly (fish, discretionary foods, and sugar-sweetened beverages), a non-significant tendency to lower odds of consuming sugar-sweetened beverages from baseline to follow-up (OR = 0.512; 95% CI: 0.261–1.003; *p* = 0.0510) was seen among FOODcamp participants compared to control participants. In conclusion, this study found no effect of the educational intervention FOODcamp on the dietary intake of vegetables, fruit, vegetable/fruit/juice combined, meat, fish, or sugar-sweetened beverages. The intake frequency of sugar-sweetened beverages tended to decrease among FOODcamp participants.

## 1. Introduction

The dietary habits of Danish children as well as children across Europe do not meet the official dietary recommendations [1,2,3]. During childhood, adherence to a healthy diet provides optimal growth and development, but it may also influence dietary practices and behaviours into adulthood [4,5]. Evidence indicates further that healthy dietary habits play an important role in the prevention of major chronic diseases in adults [6]. In the dietary recommendations, a shift towards a more plant-based diet has been a major focus during the last decade as it has been considered beneficial from both a climate and health perspective [7,8]. Based on the global EAT-Lancet reference diet [9], a nationally adapted healthy and sustainable diet was developed in Denmark in 2020 [10]. This diet recommends eating less meat (especially beef and lamb meat, both of which have a heavy environmental footprint) and substituting these protein sources with legumes and fish, and further increasing the intake of wholegrains, fruit, and vegetables, and drinking water instead of sugar-sweetened alternatives [11].

The school environment is an excellent setting to promote healthy dietary habits because it provides the potential to reach all children regardless of their socio-economic or ethnic backgrounds. School-based educational interventions focusing on healthy foods and dietary habits may include a wide range of activities such as school gardening, taste testing, and cooking classes [12]. Some of these school-based interventions have been shown to improve the dietary intake of fruit and vegetables, unhealthy snacks, and sugary drinks [13,14,15]. However, studies that also focus on the role of meat as part of a sustainable diet and the importance of decreasing meat intake are lacking. Health promotion strategies in the school setting are therefore of high relevance to develop and implement to improve the dietary habits among children and adolescents from a climate perspective. Interventions that have been considered to have a health promoting impact on the dietary habits of children and adolescents are those that include a multicomponent approach in terms of teaching content combined with experiential activities [16], and those that are implemented across multiple settings including the school, home, and/or community [17,18].

School-based food programs and education are important not only to improve the nutritional quality of children’s diets, but also to develop schoolchildren’s food literacy competencies in order for them to make informed food choices [19]. Benn 2014 defined food literacy as a broader understanding of food that does not just include nutritional knowledge and practical cooking skills but also the development of critical skills, self attributes, and competencies related to the personal self as well as one’s surroundings [19,20]. In 2014, Arla Fonden developed a 5-day educational school camp, FOODcamp (in Danish MADlejr), for children in 6th and 7th grade (aged 11–13 years). The objective of FOODcamp is to increase the children’s joy of cooking and their knowledge about healthy foods and dietary habits by engaging them with food and meals as active participants. FOODcamp is based on the official Danish dietary recommendations and provides a learning course for children where food, meals, health, and nature are addressed. The effect of FOODcamp on food literacy among Danish schoolchildren has recently been reported and a positive effect was found [21]. However, it is unclear whether improving these competencies leads to healthier dietary habits. The aim of the present study was to evaluate the effect of FOODcamp on dietary habits among 6th–7th graders, focusing on the intake of the following food groups: fruits and vegetables, fish, meat, snacks such as chips, candy, and chocolate, and sugar-sweetened beverages. We hypothesized that an increased focus on healthy dietary habits and improving food literacy would lead to behavioural changes and healthier food choices.

## 2. Materials and Methods

### 2.1. Study Design and Study Population

The effect of attending FOODcamp on dietary habits was studied in a cluster-based quasi-experimental controlled intervention study among schoolchildren from the 6th and 7th grades, aged 11–13 years. The inclusion criteria were defined by the National Food Institute (Technical University of Denmark) and Steno Diabetes Center Copenhagen. All school classes participating in FOODcamp during the school year 2019–2020 were identified by Arla Fonden. Among these, the intervention classes were chosen and the ratio between private/public schools was preferred not to exceed 1:3, which is the national ratio in Denmark, and, further, an equal distribution of 6th and 7th grades was preferred if feasible. Control classes consisted of 6th and 7th grades from the same school that were not participating in FOODcamp during the same school year, and, furthermore, had not previously participated in FOODcamp. A 6th grade class could be the control for a 7th grade class, and vice versa. In addition, a 5th grade could be the control for a 6th grade if there was no 7th grade that could act as the control (as was the case at one school). For their commitment, all participating classes received a contribution of 1000 DDK (corresponding to 134 EUR).

The schools selected to participate in the study were contacted by mail and phone and all children received an invitation letter that informed the children and their parents about the two periods with dietary registrations (before and after the intervention classes participated in FOODcamp). The participating children were recruited from nine schools (including three private schools and six public schools) located in different regions of Denmark including urban, suburban, and rural settings. In total, 267 children from 16 control classes and 322 children from 16 intervention classes were eligible (having parental consent) to participate in the study.

### 2.2. The FOODcamp Intervention

Arla Fonden provides the school food camp (FOODcamp) at two different locations in Denmark where 6th–7th graders can take part in a 5-day educational program that involves the children in different activities in order to promote healthy dietary habits in the long-term. The activities are designed to help them to develop cooking skills and to improve their understanding of the interconnection between food, health, well-being, and nature. FOODcamp has a strong focus on sustainability and different food groups (vegetables, fish, egg, and poultry), and a whole day is dedicated to food leftovers from the FOODcamp week to address food waste.

The FOODcamp intervention is based on a multicomponent intervention approach and includes educational activities that are organised in three sequential phases: (1) BEFORE FOODcamp, a school component where the topics of food, health, and nature are on the agenda and teaching takes place in the classroom at the school; (2) DURING FOODcamp, a school component where the children work with food, commodities, health, and sustainability through several different activities for 5 days at the camp; and (3) AFTER FOODcamp, a family component where the children continue working with the themes through a challenge upon returning from FOODcamp (e.g., inviting their parents for dinner in the school setting). The FOODcamp activities are described in detail elsewhere [21].

### 2.3. Background Information

Background information on the participating schoolchildren was collected with the Food Literacy Questionnaire for schoolchildren (FLQ-sc) [20], including sex, school, class, and grade.

### 2.4. Dietary Assessment

A self-administered web-based dietary record tool (the Web-based Dietary Assessment Software for Children (WebDASC)) was used for dietary intake registration. The WebDASC was developed and validated for children aged 8–11 years during the OPUS study [22,23]. In the present study, the children were asked to register their food intake on four consecutive days (Wednesday to Saturday), one to seven weeks before (baseline) and two to five weeks after (follow-up) they attended FOODcamp. The four consecutive days were selected to cover the food intake variation over days representing weekdays and weekend days, respectively. Matched intervention and control classes performed the registrations in parallel in the same week.

The WebDASC software was constructed according to the typical Danish meal pattern (i.e., breakfast, lunch, and dinner, and three in-between meals per day) and food items were categorized according to food groups. For each meal, the participants could search for food and choose pre-coded response options including food items and beverages commonly consumed in Denmark with the possibility of adding additional items in an open field. The amount consumed was registered by pointing out the closest portion size among four images from a photograph series. In addition, the tool included internal checks for frequently forgotten foods, such as spreads, sauces, snacks, candy, and beverages. After recording a whole day, the child was asked if the day represented a usual or unusual day and if there were any reasons for unusual intake, e.g., birthdays or illness. Since the schools joined the study continuously, the first dietary assessment started in August 2019 and data collection ended in October 2020. Data collection was initially scheduled to end by June 2020, but, due to the COVID-19 pandemic, the period was prolonged until the end of October 2020 in order to complete the planned data collection.

A pilot study including two classes (a 6th and a 7th grade class) was performed in order to test the practicality of a newly developed app and the WebDASC in the real-life school setting. Only 9 out of 113 participants (8%) in the pilot study were acceptable reporters, classified by cut-offs suggested by Black [24]. Therefore, actions were taken in the main study to expand communication with the participating schools and teachers compared to the pilot study. All children were instructed by study personnel, at baseline as well as at follow-up, on how to register their food and beverage intake. Depending on their need for supervision, the study personnel was present in the class on two to three weekdays during each dietary registration period (baseline and follow-up). Additionally, the teachers were instructed to remind the children to register their dietary intake every day. Since the last registration day was during the weekend, children were reminded by their teacher the following Monday and were allowed to complete their registrations during school hours if needed.

The primary outcome was the change in intake of fruit and vegetables. Secondary outcomes were the changes in intake of selected foods and food groups: vegetables/fruit/juice combined, meat (four-legged animals and poultry), fish, discretionary foods in total (chocolate, candy, and chips), and sugar-sweetened beverages.

The daily energy intake and the food groups of interest (fruits, vegetables, total fruits/vegetables/juice, meat, fish, discretionary foods, and sugar-sweetened beverages) were estimated for each participant as an average intake per day and by 10 MJ using the software system General Intake Estimation System (GIES) version 1.000i6 and the Danish Food Composition Databank version 7.0, both developed at the National Food Institute, the Technical University of Denmark [25]. Individuals with at least three and maximum five days of valid recording with daily energy intakes between 2075 kJ and 26,473 kJ for boys and 1076 kJ and 21,935 kJ for girls at baseline and at follow-up were included in the statistical analyses. This interval was based on the minimum and maximum average intake per day registered for children aged 10–13 years (N = 269) in the Danish National Survey of Diet and Physical Activity (DANSDA) 2011–2013 [1].

### 2.5. Under-, Acceptable-, and Over-Reporters

Misreporters of dietary intake in the present study were identified by evaluating reported energy against the presumed energy requirement, using the Goldberg cut-offs for the ratio between reported energy intake (EI) and estimated basal metabolic rate (BMR) at the individual level, as suggested by Black [24]. Under-reporters (UR), acceptable-reporters (AR), and over-reporters (OR) were determined by the subjects’ EI/BMR ratio. Without the weight of the children or their physical activity level, the average BMR and physical activity level (PAL) values for children aged 11–13 years from NNR 2012 were used [26]. The interval for AR was chosen to be as broad as possible and the following ranges were used to define UR, AR, and OR, respectively. UR: EI/BMR ≤ 1.097 (low level of activity, PAL = 1.66 (10–18 y)), AR: 1.097 < EI/BMR < 2.799, and OR: 2.799 ≤ EI/BMR (high level of activity, PAL = 1.85 (10–18 y)).

### 2.6. Statistical Analysis

SAS version SAS 9.4M5 was used for all statistical analyses. The applied significance level was chosen as *p* < 0.05. Model fit was checked by residual plots including QQ (quantile-quantile) plots. The outcomes were all continuous variables, and all outcomes were transformed using the logarithm (log_e_).

The effect of the FOODcamp intervention compared with the controls was investigated for each food group using constrained hierarchical mixed models [27,28]. The models included three random effects (schools, classes nested within schools, and children nested within classes and schools). The models also included the following fixed effects: sex (boy/girl), period (baseline/follow-up), as well as the effect of FOODcamp intervention compared with the controls at follow-up. For the three food groups (1) fish, (2) discretionary foods, and (3) sugar-sweetened beverages, more than a few children had zero intake, implying a semi-continuous outcome. The modelling was therefore performed in two steps [29]: first, a logistic model for the binary outcome (intake vs. no intake) giving the odds of having a non-zero intake (model 1), and second, a mixed model based on the normal distribution for children with a positive intake only (model 2). Both model 1 and model 2 are constrained, hierarchical, mixed models as described above.

When model 2 was applied, all children with at least one positive intake (i.e., an average intake over 3–5 days of the specific food) at either baseline and/or follow-up were included in the analysis (model 2) of the specific food. For the food groups, fruits, vegetables, vegetables/fruit/juice combined, and meat (four-legged animals and poultry), only model 2 was applied, since almost all children had such a positive intake. The number of children who contributed to the analysis in the control group and in the intervention group at, respectively, baseline and follow-up are presented in Table 1. As each food intake in the analyses is the average of the food intake over 3–5 days, a robustness analysis was made where the inverse variance on the averages was used as a weight. Introducing weights in the analyses did not change the results.

Intake data were initially analysed under the parallel group assumption. The analysis showed that the difference in intake between the intervention and the control group at baseline was not statistically significant for any of the food groups. The recruited schools in the study were diverse (different size, public/private, urban/rural) and geographically covered different parts of Denmark. Under the assumption that the pairing of each intervention class with a control class within the same school caused the control and the intervention group not to differ at baseline, the constrained models were fitted. The results for the initial models were similar to the results for the constrained models.

A power calculation was conducted to determine the minimum sample size required, identifying a 20% increase in vegetable and fruit intake (corresponding to 41 g/day), which is similar to the level used in other studies [13,30]. The calculation is based on 80% power and a significance level of 5%. In order to take into account the smaller variation that might exist within clusters, an intra-cluster correlation of 0.01 was used [13,30]. In addition, a dropout rate of 25% was expected based on findings from earlier intervention studies [31,32,33]. According to the power calculation, at least 12 intervention classes and 12 control classes should be recruited with at least 20 children in each class (in total 480 children).

## 3. Results

### 3.1. Study Population

**Figure 1:**
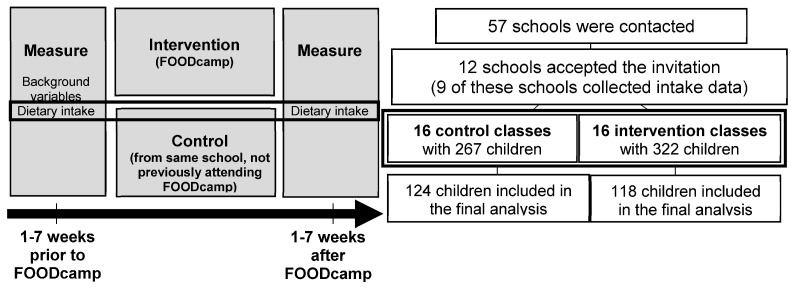
Flowchart of study design and recruitment of participants.

The flowchart of the study design and recruitment of participants is shown in Figure 1. Fifty-seven primary schools were contacted and invited to participate in the study. Of these, 12 schools accepted the invitation and were included. However, due to the COVID-19 pandemic, three schools were excluded, resulting in nine schools participating in the intervention study. In total, 589 children from 16 control and 16 intervention classes had parental consent to participate in the study. Intake data from children with incomplete dietary intake recordings were excluded, leaving in total 242 children with eligible dietary intake registrations for 3–5 days, as well as at baseline and follow-up (124 children from the control classes and 118 children from the intervention classes). The number of children who contributed to the analysis in the control group and in the intervention group at, respectively, baseline and follow-up is presented in Table 2.

In the dietary assessment, 70% of the children were classified as AR at baseline. This number decreased to 57% at follow-up. One child was classified as OR at follow-up, resulting in 30% and 43% of the children being classified as UR at baseline and follow-up, respectively.

### 3.2. Intake of Selected Food Groups

Of the seven selected food groups (vegetables, fruit, vegetable/fruit/juice combined, meat, fish, discretionary foods, and sugar-sweetened beverages), three were not eaten regularly (Table 1). For these three food groups (fish, discretionary foods, and sugar-sweetened beverages), the numbers of children with a zero intake in the control and intervention groups at baseline were 66, 7, 17 and 63, 8, 27, respectively. At follow-up, the number of children with a zero intake had increased for all food groups except fish in the control group (60, 15, 34 and 68, 19, 49, respectively).

The median (10th percentile, 90th percentile) daily intakes of the selected food groups in the control and intervention groups at baseline and follow-up are presented in Table 1. For most food groups, a decrease in the median food intake in the control group as well as in the intervention group from baseline to follow-up was observed (Table 1). Median energy intake decreased both in the control group (from 6.4 to 5.9 MJ/day) and in the intervention group (from 6.7 to 5.9 MJ/day) from baseline to follow-up (data not shown). In addition, the intakes of different food groups were energy adjusted (g/10 MJ) on an individual level, but this did not change the results, as described below (data not shown).

### 3.3. Effects of FOODcamp on the Intake of Selected Food Groups

No statistically significant effects of participating in FOODcamp were found on the average food intake of the four food groups eaten regularly (vegetables, fruit, vegetables/fruit/ juice combined, or meat) (*p* > 0.05) (Table 2).

Among the three food groups that were not eaten regularly (fish, discretionary foods, and sugar-sweetened beverages), differences were seen in the proportion of children consuming these foods from baseline to follow-up. The proportion of children in the control group consuming fish increased from 47% at baseline to 52% at follow-up, and, for the intervention group, the number decreased from 47% at baseline to 42% at follow-up. Discretionary foods were eaten by 94% in the control group at baseline and 88% at follow-up. In the intervention group, the numbers were 93% at baseline and 84% at follow-up. Finally, the percentage of children drinking sugar-sweetened beverages decreased in the control group from 86% to 73% and in the intervention group from 77% to 58%. The results are not shown but are calculated from data presented in Table 2.

Table 2 shows the odds ratios of eating the food groups not eaten regularly (fish, discretionary foods, and sugar-sweetened beverages). A non-significant tendency to lower odds of consuming sugar-sweetened beverages (odds-ratio = 0.512, *p* = 0.0510) was seen among children joining FOODcamp compared to children in the control group. The odds ratios for eating fish or discretionary foods (or not) were not statistically significant. Among consumers (children with an intake above zero), no statistically significantly differences in the reported intakes (percentage increase/decrease) of any investigated food groups were found between the intervention and the control groups (*p* > 0.05 for all).

## 4. Discussion

This cluster-based quasi-experimental controlled school intervention showed no significant effects of participating in FOODcamp on the intake of vegetables, fruit, vegetables/fruit/juice combined, or meat. In addition, no significant effects were seen on the intake of fish and more unhealthy foods such as discretionary foods and sugar-sweetened beverages. However, we found a tendency for a negative association between participating in FOODcamp and drinking sugar-sweetened beverages in the registration period, although the results were not statistically significant. Overall, we were not able to show that participating in FOODcamp may change the dietary habits of 6th and 7th graders.

In the present study, the participating children ate less vegetables, fruit, and fish than what is recommended in the Danish food-based dietary guidelines [11]. This is not surprising as similar results have been reported in other studies [34,35]. However, the intakes of energy, vegetables, fruit, vegetable/fruit/juice combined, meat, fish, and sugar-sweetened beverages are lower in our study (between 18 and 50%) than reported for the same age group in the general Danish population in the latest national survey (DANSDA) from 2011 to 2013 [1], while the intake of discretionary foods is higher (between 10 and 27%). A possible explanation could be that the dietary habits in this age group have been impaired in general during the recent years [36]. The fact that the study partly took place during the COVID-19 pandemic, when more time was spent at home, might have resulted in the increased snacking of discretionary food and drinks. Future national surveys conducted in the general Danish population may confirm whether this is the case.

Dietary habits take a long time to change [37], probably a longer time than the duration of the FOODcamp educational program. In addition, parental involvement is important since children aged 11–13 years share eating habits with their families. Elsborg et al. found in the same study population a small but significant effect of FOODCamp on children’s food literacy, where both overall food literacy and the three food literacy competencies “to do”, “to sense”, and “to know” were significantly increased [21]. We could not show that FOODcamp changed the participating children’s dietary habits. Even though one of the topics of FOODcamp is the role of meat as part of a sustainable diet and reducing meat intake, this was not reflected by a decrease in meat intake among participating children as expected. One explanation could be that dietary changes are the very last thing that follows changes in children’s food literacy competencies. Another explanation could be that food choice and eating behaviour in children are probably affected by multiple levels of interacting factors including individual factors (e.g., cognitions, gender, age, taste preferences) and social environmental factors (e.g., interactions with family and friends) [38]. Finally, it cannot be ruled out that the intervention period was too short, in combination with the fact that children aged 11–13 years are not the main ones responsible for the food served at home. Therefore, it could be interesting to further include activities involving the families, and repeat the dietary assessment to track any long-term changes in dietary behaviour. School-based intervention studies are considered successful specifically if they include parental involvement and multiple strategies to assure an impact on the long-term behaviour [12,39].

A direct comparison with other studies is difficult due to differences in study designs and dietary assessment methodologies. Our study found no effects of participating in FOODcamp on dietary habits among 6th and 7th graders. This is in agreement with some of the findings of a previous cooking and nutrition education intervention by Jarpe-Ratner and co-authors among 3rd to 8th graders in the US [40]. They reported no statistically significant effect on the intake of chips or soda, which is similar to our findings, but they showed improvements in fruit and vegetable intake [40]. In the present study, we focused on different aspects of healthy dietary habits and hands-on cooking activities during a five-day educational school camp. In the study by Jarpe-Ratner and co-authors, the main focus was on fruits and vegetables as part of a well-balanced meal during a ten-week after-school education course (one session per week). Furthermore, they involved the families, as they were encouraged to discuss healthy diets and cooking with their parents at home [40]. The increased attention on fruits and vegetables, and family involvement, could explain the different findings on fruit and vegetable intake. Murimi and co-authors [37] prepared a systematic review focusing on factors contributing to effective nutrition education among children. They concluded that successful interventions in elementary school should include parental engagement in order to have specific and measurable behavioural outcomes, and the adequate duration of an intervention (at least 6 months) and age-appropriate activities are important. These results may indicate that school-based interventions alone cannot change behaviour, since the interaction with parents and other family members is crucial. The educational program of FOODcamp targeted the relevant age group, but involved the parents to a lesser extent due to the COVID-19 pandemic, as no planned activities involving parents were carried out. This might have affected the results in the present study. 

Collecting dietary data from children is challenging, and recall problems and under-reporting are the main challenges. Furthermore, the estimation of portion sizes is difficult for many children, and even adults struggle with this aspect [41]. Based on the results from the pilot study where the number of acceptable reporters was very low, actions were taken to strengthen communication with the schools and teachers in this intervention study. Therefore, the dietary registration was performed individually in the class during school lessons (on at least two days of the four-day registration period at baseline as well as at follow-up). Moreover, the children’s work and their effort to register their diet was acknowledged by a financial donation to all participating classes. Even though these measures were labour-intensive, they improved the data collection considerably compared to the pilot study.

The present study was aimed at collecting data during a four-day dietary registration period, since it is a strength that the dietary registration includes one weekend day (Saturday) and at least three weekdays. Nordman et al. found that especially among children, dietary intake fluctuates substantially during the week, with weekend days having lower dietary quality than weekdays [39]. In our study, some children registered their intake on five days, even though three days of registration were considered acceptable. It is a limitation that many children had dietary registrations of low quality in terms of too few days or incomplete registrations. Only 36% of the children in the intervention group and 46% in the control group had eligible dietary intake recording to be included in the analyses. Another limitation in the present study, as in dietary surveys in general, is that participants may register a diet that is healthier than the diet they have actually eaten, leading to the misclassification of dietary intake estimates due to social desirability bias [42]. In addition, children in the under-reporters group increased from 30% at baseline to 43% at follow-up. Other studies found 25–30% to be under-reporters [43,44]. The under- and over-reporting of energy intake might be a bias that could affect the results of the dietary assessment. This study has the same level of under- and over-reporters in both the control group and the intervention group, indicating no distortion of the results between the groups. Finally, a lack of randomization is a limitation of the present study. FOODcamp is an ongoing educational program where school classes apply for participation and are enrolled continuously, which implies that randomization is not possible.

## 5. Conclusions

In conclusion, this study found no effect of the educational intervention FOODcamp on the dietary intake of selected food groups (vegetables, fruit, vegetable/fruit/juice combined, meat, fish, or sugar-sweetened beverages). The intake frequency of sugar-sweetened beverages tended to decrease among FOODcamp participants compared with controls, but not significantly. We cannot exclude that dietary behaviour is affected later in life when the children become independent of their parents and responsible for their own cooking.

## Figures and Tables

**Table 1:** Estimated daily dietary intake in children at baseline and at follow-up for control and intervention groups (median and percentiles).

	BASELINE (n = 242)								FOLLOW-UP (n = 242)							
	Control Group (n = 124)				Intervention Group (n = 118)				Control Group (n = 124)				Intervention Group (n = 118)			
		Percentiles				Percentiles				Percentiles				Percentiles		
Food group (g/d)	n_0_	Median	P10	P90	n_0_	Median	P10	P90	n_0_	Median	P10	P90	n_0_	Median	P10	P90
Vegetables	1	94	35	215	0	115	31	284	1	90	24	193	3	92	36	224
Fruit	3	83	6	268	4	90	14	315	8	73	1.2	198	10	77	0.2	239
Vegetables, Fruit, and Juice	0	213	82	483	0	243	99	503	0	185	60	376	0	197	64	459
Meat	2	80	35	178	4	93	22	209	2	81	24	165	4	81	16	195
Fish	66	0	0	29	63	0	0	41	60	2	0	39	68	0	0	34
Chocolate, Candy, and Chips	7	27	5	94	8	31	1	87	15	25	0	77	19	23	0	84
Sugar-sweetened beverages	17	133	0	474	27	91	0	382	34	68	0	315	49	18	0	420

n_0_: Number of children with zero intake (percentiles include children with zero intake).

**Table 2:** Odds ratio of non-zero intake for the intervention group compared with the control group for the three food groups “Fish”, “Chips, Candy and Chocolate” and “Sugar-sweetened beverages” (model 1), and the effect of the intervention on the intake of all food groups compared with control (model 2) (odds ratios, estimates, and 95% confidence intervals).

				Model 1 *, All Children			Model 2 ^†^, Children with Intake > 0		
Food Group	n_c(total)_/n_i(total)_	n_c_/n_i_ Baseline	n_c_/n_i_ Follow-Up	*p*	OR	95% CI	*p*	Estimate ^ǂ^	95% CI ^ǂ^
Vegetables	124/118	123/118	123/115	-	-	-	0.5192	0.05776	−0.1185, 0.234
Fruit	124/118	121/114	116/108	-	-	-	0.4839	−0.1286	−0.4903, 0.2331
Vegetables, Fruit, and Juice	124/118	124/118	124/118	-	-	-	0.7311	−0.03036	−0.2042, 0.1435
Meat	124/118	122/114	122/114	-	-	-	0.3390	−0.09616	−0.2939, 0.1016
Fish	124/118	58/55	64/50	0.0788	0.587	0.324, 1.063	0.2837	0.2412	−0.2024, 0.6848
Chips, candy, and chocolate	124/118	117/110	109/99	0.8211	0.832	0.169, 4.103	0.7244	−0.05368	−0.3535, 0.2461
Sugar-sweetened beverages	124/118	107/91	90/69	0.0510	0.512	0.261, 1.003	0.4448	0.1380	−0.2181, 0.4942

n_c (total)_: total number of children in the control group. n_i (total)_: total number of children in the intervention group. n_c_: number of children in the control group with an intake above zero. n_i_: number of children in the intervention group with an intake above zero. OR: odds ratio. * Model 1 used on the three food groups (“Fish”, “Chips, Candy and Chocolate”, and “Sugar-sweetened beverages”) where more than a few children have zero intakes, analysed by a logistic regression model for the binary outcome (intake vs. no intake) giving the odds of having a non-zero intake for the intervention group compared with the control group. ^†^ Model 2 includes children with intakes above zero, analysed by constrained hierarchical mixed models, including random effects (school, class, and child) and fixed effects (sex, period, and effect of intervention compared to controls at follow-up). ^ǂ^ Intake data from all food groups were log_e_ transformed and therefore estimates are expressed as percentages (includes children with intakes above zero). As an example, the non-significant estimate of 0.05776 for vegetables (model 2) means that the intake in the intervention group is 5.8% higher compared to the intake in the control group.

## Data Availability

Collected and analysed data in the present study are available upon reasonable request to the corresponding author.
